# The Atlanta Medical Society

**Published:** 1883-01

**Authors:** 


### THE ATLANTA MEDICAL SOCIETY.

*Reported for the Atlanta Medical Register.*

ATLANTA, November, 1882.

The President, Dr. W. S. Armstrong, in the chair.

Dr. A. W. Calhoun, referring to an epidemic of sore eyes that prevailed throughout large sections of the country, said the disease was really purulent ophthalmia. It is the most severe epidemic of this disorder he has ever known, and extends over the Northern and Southern States. In Georgia and Alabama it is exceptionally bad, and the results are exceedingly fatal to vision from ulceration of the cornea. When the ulceration is central—that is, situated over the pupil—blindness ensues, whether the ulceration is large or small, from opacity of the corneal tissues. In a large number of cases the cornea sloughs off. He has done many operations for stapheloma and removal of the ball in consequence of this disease in children. A remarkable exemption of the negro race has been observed. Out of hundreds of cases among the whites, only one or two cases have been known to occur among the negroes.

In the treatment, absolute cleanliness is essential. When the lids are much swollen, it sometimes becomes necessary to split up the external canthus in order to take the pressure off of the ball. The bleeding, after this operation, is profuse on account of the congestion of the parts, and the local depletion is favorable. Of course, this procedure is only applicable when the lids are intensely swollen and cannot be opened, and when they press dangerously upon the cornea. Thus cut to the orbital margin, no harm can result from the simple operation, even should it be shown that it was not absolutely necessary.

As to medication, the best application is a solution of nitrate of silver—grs. x, ℥j—but in infants the excess should be immediately neutralized by salt water. In adults the strength of the silver solution should be the same, but the salt is not necessary. The duration of the disease is two or three weeks, and this application should be made once a day. At the same time a few drops of a solution containing two grains each of alum, morphine and sulphate of zinc to the ounce of water should be dropped into the eye two or three times daily.

The amount of pus secreted under the influence of this disease is enormous. In adults it is sometimes as much as a tea-cup full from each eye in twenty-four hours. He is unable to account satisfactorily for the present epidemic. An old gentleman of his acquaintance suggested that it was due to the unusual number of gnats, which he believed conveyed the poison from infected to well individuals.

The disease has been so prevalent in Illinois that the State Board of Health took up the subject and offered suggestions as to its management.

It is a popular belief that sore eyes may be contracted by simply looking at an affected individual. Of course, there is nothing in that notion. The disease can only spread by contact or by its peculiar infectious influence. It is the same disease—excepting the specific cause—and almost as severe, as the true gonorrhœal ophthalmia. All gonorrhœal ophthalmia is purulent ophthalmia but purulent ophthalmia is not necessarily gonorrhœal. One attack does not afford immunity from another.

Dr. James B. Baird said he had several times resorted to a simple device for elevating the lids in this disease. This consisted in doubling over the convex or rounded end of an ordinary hair-pin so as to form a blunt hook. A

little experience will suggest the proper angle to give the hook, and when carefully used it affords admirable facility for the escape of imprisoned secretions under the lid and for the application of medicated solutions. Care should be observed in the employment of the hook, but the expedient is serviceable in the absence of a specifically constructed elevator. Any manipulation of this kind is rendered much easier by first rolling the child in a shawl so as to confine its arms and legs.

Dr. Calhoun added that great advantage was gained by holding the head of the child fast between the knees of the operator. He regarded the suggestion of Dr. Baird about the improvised elevators a good one. It renders the sulcus between the lid and the ball accessible. There is, however, great danger that the thin cornea may be ruptured by the slightest violence. Once in a hospital he saw the distinguished Arlt rupture the cornea and allow the lens to escape. It is an unfortunate accident.

Dr. Calhoun, continuing, remarked that he had recently seen two interesting cases of Bright's disease affecting the eye. The first was in the person of a young lady who became suddenly blind in one eye. She developed acute optic neuritis and had frequent retinal hemorrhages. In a few days the other eye was similarly affected. Before death she was almost totally blind. Medication is unsatisfactory.

Another case occurred in a stout man who had frequent retinal hemorrhage. Tests applied to his urine revealed the existence of Bright's disease. He gave an unfavorable prognosis. He is informed that the man is growing progressively weaker. The only treatment of any value is the administration of general tonics. If no fresh hemorrhages occur the clots may be absorbed and vision gradually be improved ; but this result is scarcely to be hoped for, as other hemorrhages are almost certain to take place, one after another, as the general disease progresses.

Dr. G. G. Roy remarked that Bright's disease is so insidious in its development that both kidneys and the eye, when it is involved, are hopelessly injured before treatment is sought for the eye trouble. More, in the way of treatment, might be accomplished, if it could be earlier applied.

Dr. Baird reported a case of acute articular rheumatism in a gentleman aged about sixty years. Most of the larger joints were affected, and his suffering was intense. He prescribed salicylic acid in combination with acetate of potash and sweet spirits of nitre, with the happiest results. Profuse diaphoresis and diuresis promptly followed its administration, and the heat, swelling and pain around the joints rapidly subsided. Every dose represented fifteen grains each of the acid and the potash salt, and half a drachm to one drachm of the sweet spirits of nitre, given every six hours. This combination, in about one drachm and a half of water, makes a clear solution. Of course it should be administered largely diluted. The stomach of his patient did not rebel at the remedy, as is frequently the case—though he thinks the addition of the alkali renders the acid less obnoxious to most persons—but an alarming degree of prostration ensued after several doses had been taken. The pulse sunk very low, skin became cold and profusely bathed in perspiration, and mild hallucinations were experienced. Alcohol was very objectionable to the patient, so carbonate of ammonia was used, and reaction was secured. Subsequent efforts to resume the use of the drug were invariably followed by this depressed condition, but by increasing the intervals and, at the same time diminishing the quantity, with the aid of the ammonia he was enabled to keep the acute symptoms in subjection. In a short time the trouble in the larger joints had almost entirely disappeared, but several of the smaller joints of the foot and hand had become affected, accompanied by great local swelling, which seemed to contain a semi-solid, gelatinous substance. At this stage, tincture of colchicum was added to the mixture already prescribed, and it acted like a charm.

This was the first case in which he had observed anything approaching an alarming depression following the use of the salicylic acid, and he has by this experience been impressed with the importance of caution in its use. He regards it a very valuable remedy in the treatment, not only of acute, but also of subacute and chronic rheumatism. He has not infrequently employed it in the latter class of cases with results comparable to those generally secured in the acute form of the disease.

Dr. Roy said the state of depression just described was invariable if large doses of the drug were continued—sixty or eighty grains a day are almost certain to bring it about. Combined with quinine it is a remarkable and most valuable remedy. Reduction in the pulse-beat and the temperature accounts for the depression. He considers it dangerous to commence with more than ten-grain doses. As soon as nervous symptoms become manifest its administration should be stopped, or it may be difficult to secure reaction. Its continued exhibition under these circumstances produces a state bordering on death.

Dr. W. S. Armstrong regards salicylic acid as a very valuable recent addition to our therapeutic resources. Recently encountered a case of acute rheumatism in a boy. He was put on a treatment of salicylic acid and alkalies. In three days he was up and about. Still, in other cases he has been disappointed in its action. In his own personal experience, at the commencement of an acute attack, several years ago, he took it freely, and in a few days the disease was all gone. He felt well, appetite good, and he resumed his ordinary business. After several days another attack set in. The same remedy taken in the same way exerted no beneficial effect. He experienced a sensation of sinking, depression, vertigo and only very slight diaphoresis and a constant tendency toward mental wanderings. He dropped the acid. It became very offensive to him. He preferred the disease to the remedy, and the feeling of mental instability which it engendered. He has observed that where the patient tolerates the remedy and it produces abundant diaphoresis, the effect is beneficial, and under these circumstances, if used early, even if it doesn't abort the disease, it modifies its severity and proves very serviceable in that way; but if he is called late, after the full developement and some progress of the disease, he expects to obtain no good effects from it.

